# Cystic fibrosis lung environment and *Pseudomonas aeruginosa* infection

**DOI:** 10.1186/s12890-016-0339-5

**Published:** 2016-12-05

**Authors:** Anjali Y. Bhagirath, Yanqi Li, Deepti Somayajula, Maryam Dadashi, Sara Badr, Kangmin Duan

**Affiliations:** 1 Department of Oral Biology, Rady Faculty of Health Sciences, University of Manitoba, 780 Bannatyne Ave, Winnipeg, MB R3E 0W2 Canada; 2 Department of Medical Microbiology, Rady Faculty of Health Sciences, University of Manitoba, 780 Bannatyne Ave, Winnipeg, MB R3E 0W2 Canada; 3 Biology of Breathing Group, Children’s Hospital Research Institute of Manitoba, 780 Bannatyne Ave, Winnipeg, MB R3E 0W2 Canada

**Keywords:** Cystic fibrosis, Host-pathogen interaction, Microbiome, CFTR, Non-genetic influences

## Abstract

**Background:**

The airways of patients with cystic fibrosis (CF) are highly complex, subject to various environmental conditions as well as a distinct microbiota. *Pseudomonas aeruginosa* is recognized as one of the most important pulmonary pathogens and the predominant cause of morbidity and mortality in CF. A multifarious interplay between the host, pathogens, microbiota, and the environment shapes the course of the disease. There have been several excellent reviews detailing CF pathology, *Pseudomonas* and the role of environment in CF but only a few reviews connect these entities with regards to influence on the overall course of the disease. A holistic understanding of contributing factors is pertinent to inform new research and therapeutics.

**Discussion:**

In this article, we discuss the deterministic alterations in lung physiology as a result of CF. We also revisit the impact of those changes on the microbiota, with special emphasis on *P. aeruginosa* and the influence of other non-genetic factors on CF. Substantial past and current research on various genetic and non-genetic aspects of cystic fibrosis has been reviewed to assess the effect of different factors on CF pulmonary infection. A thorough review of contributing factors in CF and the alterations in lung physiology indicate that CF lung infection is multi-factorial with no isolated cause that should be solely targeted to control disease progression. A combinatorial approach may be required to ensure better disease outcomes.

**Conclusion:**

CF lung infection is a complex disease and requires a broad multidisciplinary approach to improve CF disease outcomes. A holistic understanding of the underlying mechanisms and non-genetic contributing factors in CF is central to development of new and targeted therapeutic strategies.

## Background

Cystic fibrosis (CF) is the most common life-threatening autosomal recessive genetic disease in Caucasians. The estimated incidence of CF is one in 2500–4000 within the Caucasian population and holds a prevalence of about 100,000 globally [1]. Fortunately, the incidence rate and overall prevalence for CF has been declining [2] over the recent years. This is a result of neonatal screening and newer treatment modalities such as improved control of pulmonary infections and mucociliary clearance. However, recent years have also seen an increase in disease complexity [3] with newer, more resistant genetic variants [4] often emerging. Some of these have been covered very well in several excellent reviews [3, 5–7]. One common theme that gets less than deserved attention is the interplay of immediate environmental factors in shaping the course of disease progression. We believe that a thorough understanding of the environmental factors can help in defining the course of the disease. With such knowledge, a more appropriate and patient-specific treatment approach can be taken. Though there are several excellent reviews that cover aspects of CF and CF lung environment, this article aims to provide a comprehensive review on recent knowledge on the influence of the lung environment in shaping the disease in a holistic manner.

## Discussion

### The pathophysiology of cystic fibrosis

CF is caused by mutations in the cystic fibrosis transmembrane conductance regulator [CFTR] gene. Currently, more than 2000 mutations have been identified, of which 127 are confirmed as disease-causing [8]. However, the molecular mechanisms underlying the strict transcriptional regulation of CFTRs remain poorly understood. CFTR/ABCC7 is a cyclic adenosine monophosphate (cAMP)-dependent member of the adenosine triphosphate (ATP)-binding cassette transporter super family, found in the apical membrane of epithelial cells. It is the only member of the ATP-binding cassette protein family known to function as an ion channel rather than as an active transporter. CFTRs are expressed in many organs such as the kidneys, pancreas, intestine, heart, vas deferens and lungs [9]. CFTRs have been shown to perform a significant role in regulation of sodium [10–12], potassium [13–15], outward rectifying chloride channels [16, 17], calcium-activated chloride channels [18, 19], sodium bicarbonate [20, 21] and aquaporin [22] channels. Other CFTR functions include the regulation of vesicle trafficking, ATP release, and the expression of inflammatory epithelial mediators (Interleukin 8 and 10, and inducible nitric oxide synthase) [23]. These findings link the complex and diverse CFTR functions to CF lung disease.

**Fig. 1:**
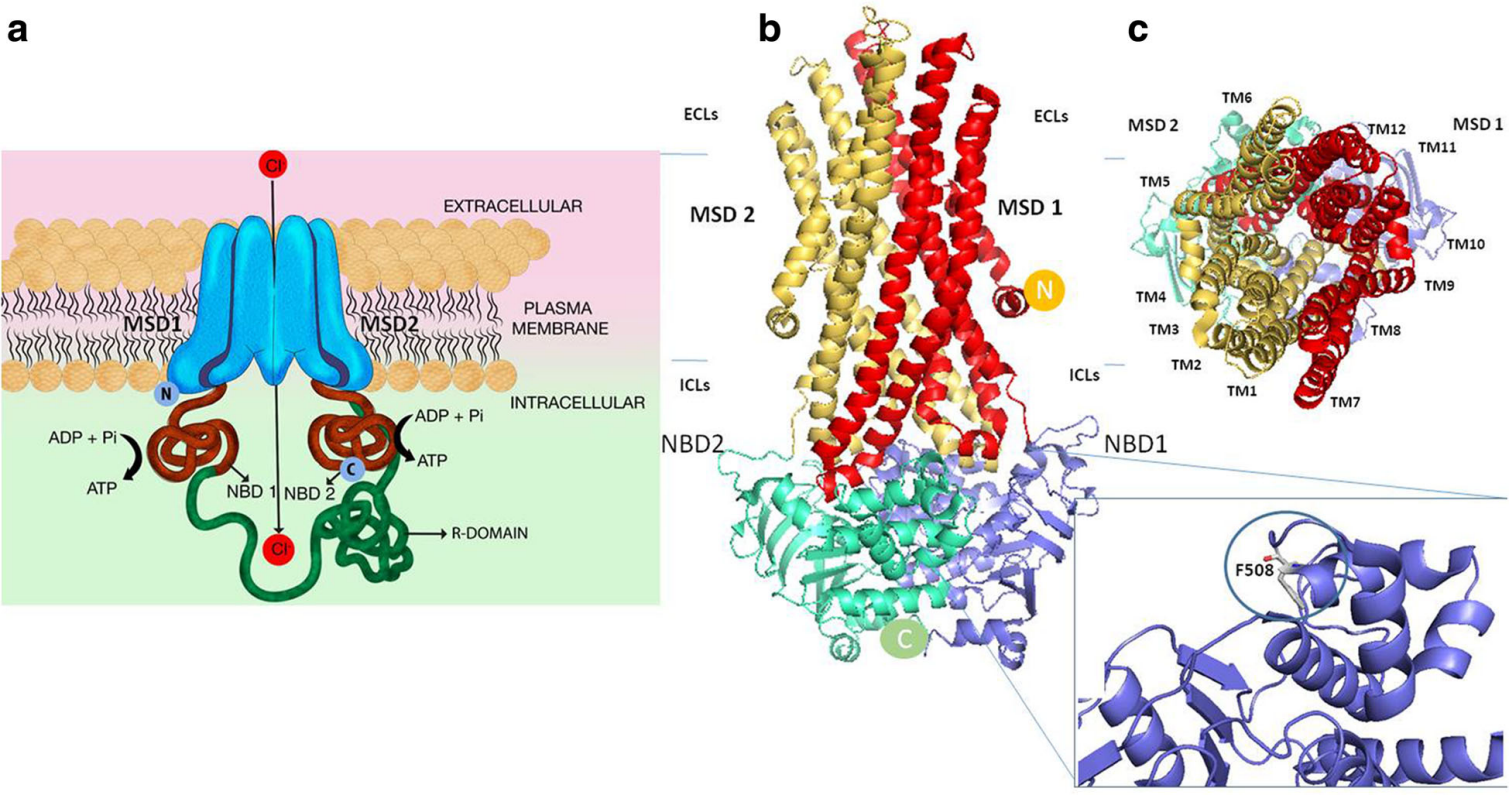
Two-dimensional representation of CFTR channel and homology models. **a** A cartoon representation of CFTR. CFTR is composed of two membrane spanning domains (MSDs), each linked through intracellular loops (ICLs) and extracellular loops (ECLs) (not shown here) to nucleotide binding domains (NBD1 and 2) (red). Unique to CFTR, NBD1 is connected to the MSD2 by a regulatory domain (R). **b** The three-dimensional homology model for CFTR based on Sav1866 structure (2HYD) [25–28]. MSD1 contains transmembrane helices (TM) 1–6 and MSD2 contains TMs 7–12. The amino N terminus and carboxyl-terminus are labelled respectively as N and C in yellow and shown circles. Insert shows F508A mutation in NBD1 crystal structure (1XMI) seen in gray [28]. **c** CFTR is shown in its outward facing (extracellular) conformation

Structurally, a CFTR is a membrane-bound glycoprotein of 1480 amino acid residues, with a molecular mass of 170,000. CFTR has a typical architecture of 12 transmembrane spanning helices arranged into two pseudo-symmetrical transmembrane domains and two nuclear-binding domains (NBDs) which bind and hydrolyze ATP and contain several highly conserved motifs (Fig. 1a) [24–28]. These two NBDs, NBD1 and NBD2, can form a “head-to-tail” dimer, forming two composite binding sites for ATP at their interface. Between the two NBD units is a unique regulatory (R) domain which is made up of many charged amino acids [29]. The loss of phenylalanine at position 508 in the CFTR gene is the most common mutation in CF and occurs in a highly conserved α-helical sub domain (495–565) in NBD1 (Fig. 1b) [30]. Similarly, many of the other identified mutations have also been observed to occur in NBD1, while relatively few occur in NBD2.

In a non-mutated CFTR, gating has been shown to be tightly coupled with ATPase cycles through NBD Dimerization, which then induces the formation of a transmembrane domain cavity that opens towards the extracellular side to allow for selective anion flow (Fig. 1c) [31–33]. Deletion of phenylalanine at 508 (F508del) in NBD1 leads to a CFTR trafficking defect. Biophysical studies of F508del in solution with “stabilizing mutations” at a second site (s) demonstrated inherent alterations in kinetic and thermal stability [34–36]. Studies also observed that Mg-ATP binding delays unfolding of the wild-type but not the mutated NBD1 [36]. Fluorescence resonance energy transfer studies however showed that F508del mutation does not impair ATP binding, rather it affects ATP-dependent interactions between the two non-contiguous regions in the core F1-ATPase domain of NBD1 [37]. It has also been shown that the intra-domain defect caused by F508del in NBD1 affects the assembly of the full-length CFTR protein and eventually the post-translational stability. This instability affects the maturation of core glycosylated F508del-CFTR to a complex glycosylated protein and leads to endoplasmic retention [37] and eventually decreased expression of the functional protein on the cell surface [29]. Thus, it was hypothesized that the defect may be corrected by improving the interaction between NBD1 and NBD2. However, interventions aimed at disrupting or enhancing the interaction of NBD1 and NBD2 have not been shown to affect the biosynthesis and processing of F508del-CFTR.

NBD-intracellular loop (ICL) interactions have been shown to be involved in ATP binding and channel gating. Studies have observed an interaction between NBD’s interface and ICL2 and ICL4 in the wild type; this interaction is altered in F508del with effect on channel gating. Interventions designed to modify the interaction between the surface on NBD1 lacking F508 and the coupling helix presented by ICL4 have been shown to significantly enhance the biosynthesis and processing of F508del-CFTR [38, 39]. Mendoza et al. [38] also described mutations in the coupling helix of ICL4 that cause ER retention (L1065P, R1066C, and G1069R), supporting the idea that this region does play a role in mediating significant interactions during folding. For details of CFTR protein function, readers are referred to articles by Borowitz, Eborn, and Meyerholz [20, 40, 41].

The current paradigm is not to view CFTR as just an ion channel, but a signaling system. It has been hypothesized that if the cellular environment can be altered, the CFTR protein defect may be bypassed. One of the ways to approach this is by improving proteostasis in CF cells [42–44]. Proteostasis improvement helps to re-establish the plasma membrane localization of CFTR. This is achieved by remodeling the F508del-CFTR interactome and avoiding unwanted interactions, thus reinstating desirable protein-protein interactions for F508del-CFTR [43]. In pigs and mice with CFTR defect, administration of proteostasis modulators such as cysteamine significantly reduced mortality, improved weight gain, and increased the expression of functional CFTR protein at the intestinal level [43].

Owing to the complex function of CFTR, multiple physiological disorders arise in CF. Dysfunctional CFTR in the secretory epithelial cells results in obstructions in the lung airways and pancreatic ducts as a major pathological consequence. In CF airways, CFTR dysfunction or absence instigates the accumulation of abnormally thick, sticky mucus in the respiratory tract, which hampers bacterial mucociliary clearance and allows the colonization of the airways by microbial pathogens (discussed further in the following sections). The most notable bacterial pathogens include *Pseudomonas aeruginosa*, *Staphylococcus aureus*, *Haemophilus influenzae* and *Burkholderia cepacia* complex, with *P. aeruginosa* causing the most predominant lung infection in CF. The dominant chronic inflammation is generated by the failure of microbial clearance and the creation of a toxic pro-inflammatory local microenvironment, which damages the lung and the innate immunity, further facilitating infections.

Normally, airway epithelial cells can ingest the invading pathogens such as *P. aeruginosa,* followed by desquamation, thus protecting lungs from injury. In CF, however it has been observed that CF epithelial cells phagocytose fewer cells of *P. aeruginosa* [45]. It was initially suggested that CFTR is a cell-surface receptor for *P. aeruginosa* with an intact lipopolysaccharide [LPS] core [45]. However, it was later understood that the internalization of *P. aeruginosa* in epithelial cells does not involve the chloride conductance channels but lipid rafts [46]. After *P. aeruginosa* enters cultured epithelial cells, the infected cells display plasma membrane blebs, while others show co-localization to acidic vacuoles [46]. An increased apoptosis has also been observed in such blebbing cells [45]. It has been suggested that the blebbing may be a response to *P. aeruginosa* LPS. Prolonged and repeated *Pseudomonas* LPS exposure in CF mice has been shown to result in abnormal and persistent immune response and significant structural changes in the lungs [46]. Murine CF macrophages with reduced autophagosome formation cause hypersecretion of IL-1β and enhanced survival of *Burkholderia cenocepacia* [43, 47–49], another co-habiting pathogen in CF lungs. Airway acidification by the abnormal CFTR function has also been shown to be a major factor that initiates host defense abnormalities and microbial colonization [50]. Although the detailed mechanisms of the high susceptibility of the CF lung to bacterial infections are not completely clear, increasing data are gradually revealing the properties of abnormal CFTR associated with the prevalent infection with *P. aeruginosa* and other pathogens.

What becomes clear, however, is that CF lung pathogenesis begins with the altered lung environment triggered by the abnormal CFTR. A major outcome of changes in lung environment is a shift of the balance between surviving microorganisms that enter the lung and the host defense mechanisms, which eventually result in conditions that favors the survival of the invading microbes together with a persistent yet ineffective immune responses.

### The host: Internal environment in cystic fibrosis

The lung environment in CF is different from that of healthy people and undergoes significant alterations over the course of a patient’s lifetime in terms of disease progression, microbial infections and the lung microbiota. The lung environment dictates host-microbe interactions which shape the course of disease. In the healthy respiratory system, the upper respiratory tract is colonized by microorganisms comprising the normal flora while the lower respiratory tract is relatively sterile due to the various host innate defenses. The presence of a microbiota and colonization of respiratory pathogens in lower respiratory tract of CF patients suggests fundamental differences between the CF airways and those of the healthy individuals. Such differences not only provide survivable conditions for the microbes but also alter the host-pathogen interaction. It is thus important to understand the internal environment in the CF lung.

#### Airway anatomical complexity as a contributor to disease

**Fig. 2:**
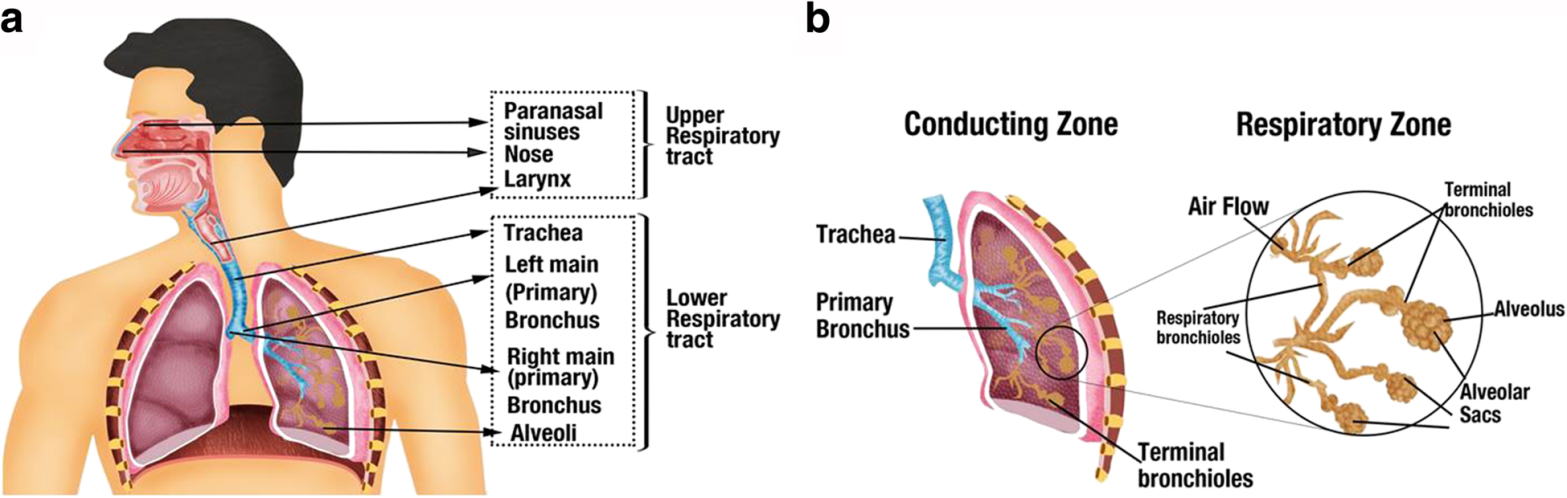
The different anatomical divisions of human respiratory system relevant to CF lung disease. Environmental factors such as oxygen and nutrient availability vary significantly in different regions of the human respiratory system and influence disease outcomes. **a** The airway can be divided primarily into the upper and lower respiratory tract. **b** Lower respiratory tract is further divided into conductive zone and the respiratory zone. The conducting zones consist of trachea, primary and terminal bronchioles. The conducting zones are secretory in function. The respiratory zones perform the function of air exchange and consist of respiratory bronchioles and alveolar sacs

The human airway is highly compartmentalized, with the upper respiratory tract, consisting of the nose and the paranasal sinuses followed by the lower respiratory tract, which is further divided into conductive and respiratory zones (Fig. 2a and b). The sinuses in the upper respiratory tract have comparatively less airflow and are more separated from antibiotic exposure and host immune responses. Their primary function is to provide resonance to sounds and produce mucus to facilitate bacterial clearance. The shape and size of the airways impacts the overall flow and resistance of air passing through them. Airway morphology is essential to lung function and has been suggested to be an indicator for disease severity in patients with respiratory disease including CF [51]. Understanding airway complexity is critical to understanding respiratory symptoms, developing ways to facilitate efficient delivery of inhaled medications, and improve mucus clearance.

Studies from animal models suggest that CF patients present with abnormalities in the size and shape of the trachea from birth [52]. Pulmonary imaging of young children with CF indicates early structural defects, even in those with normal pulmonary function test results [52]. It has been observed that the airways of infants and young children with CF have thicker walls, with higher dilation, than those of normal infants [52, 53]. The sinuses in CF form a very well-protected habitat given the complexity of the anatomy and the viscidity of mucus lining it. These make them an excellent reservoir for chronic and relapsing lower respiratory infections. It has been reported that CF patients often present with chronic rhinosinusitis [54] and sinus microbiota in CF is often considered to be predictive of pulmonary disease [54–57].

**Fig. 3:**
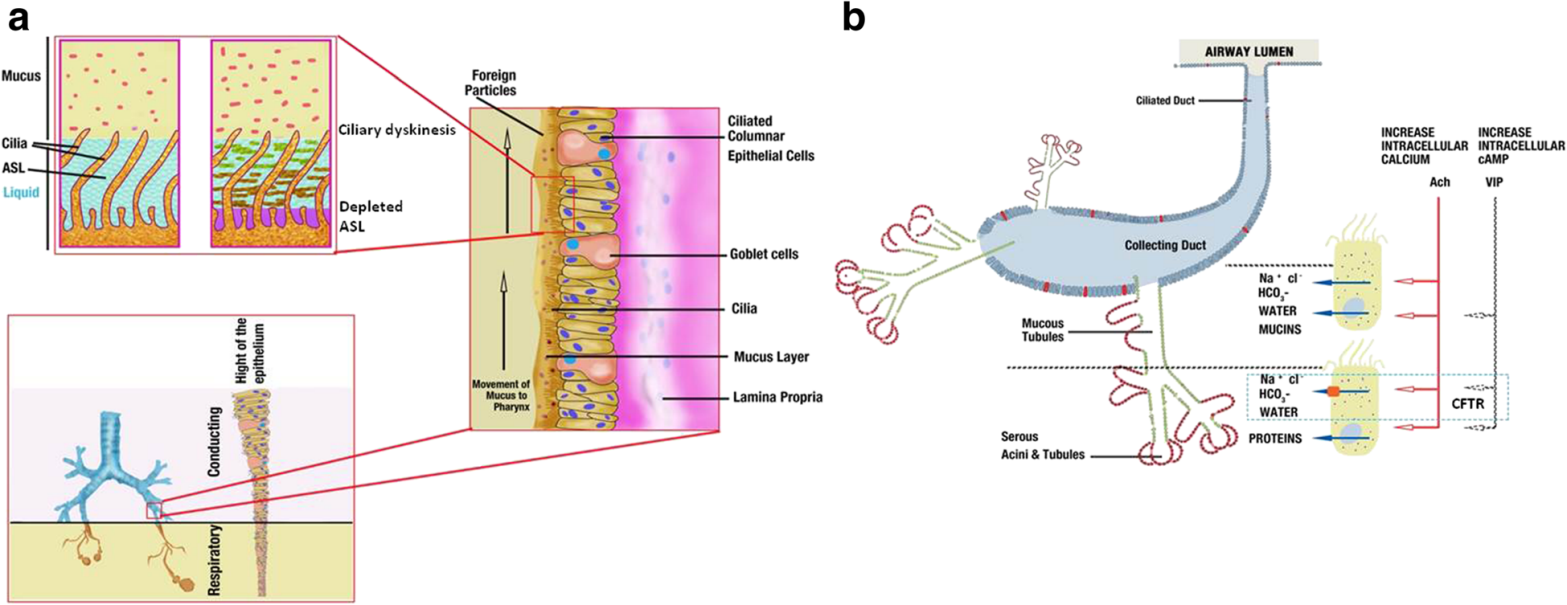
Anatomical distribution of mucus secreting cells in normal airways and pathological alterations in CF. **a** Mucus is secreted by submucosal glands in the conductive zone and paranasal sinuses. The submucosal glands go on decreasing towards the lowest components of the respiratory zone. In healthy individuals, the cilia of the epithelial cells clear irritants and microorganisms, trapping them in the thin fluidic mucus and clearing by rhythmic ciliary beating upwards known as mucous escalator. In CF, the airway surface liquid layer thins and the mucus comes in contact with cilia resulting in ciliary dyskinesis, causing poor clearance of bacteria which exacerbates inflammation. **b** Schematic drawing of a single submucosal gland shows serous acini, mucus tubules, and collecting duct. Secretion of water across the epithelium of airway glands is driven predominantly by active secretion of chloride and bicarbonate. The CFTR-dependent water-secreting pathway is defective in CF. Figure adapted from previous publications [54, 55, 57, 59]

In the lower respiratory tract, the conductive zones produce mucus and facilitate bacterial clearance, leaving the alveoli generally free of bacteria. Mucus within the conductive zones is produced by sub-mucosal glands which occur at a frequency of about 1 per mm^2^ in trachea and go down to airway lumen diameters of 1–2 mm (Fig. 3a). In healthy humans, sub-mucosal glands provide more than 95% of upper airway mucus. Each gland is composed of tubules that feed into a single collecting duct, which then narrows into a ciliated duct that is continuous with the airway surface. Tubules are lined with mucous cells in their proximal regions and serous cells in the distal acini [54, 58, 59]. Normal glands are made up of 60% serous and 40% mucous cells by volume. The serous cells secrete water, electrolytes, and a mixture of compounds with antimicrobial, anti-inflammatory and antioxidant properties, while mucous cells provide most of the mucins. Of relevance to CF is the observation that within airways, CFTRs are most highly expressed in serous cells [55–57] (Fig. 3b). Secretion of water across these glands is driven predominantly by active secretion of chloride and bicarbonate as well as increase in intracellular cAMP. Both cAMP and Cl^−^/HCO_3_^−^ can be stimulated by a variety of agonists that elevate either cAMP, Ca^2+^, or both, such as cholinergic agents and vasoactive intestinal peptide [54, 60–62]. In tracheobronchial airways of animal models, it was observed that the inhibition of Cl^−^ and HCO_3_^−^ secretion by bumetanide and dimethylamiloride in submucosal glands produces CF-like pathology, including production of thick dehydrated mucus and occlusion of gland ducts [63, 64]. The mucus secretions from the submucosal glands in lower airways are also important for mucociliary clearance and provide major antimicrobial proteins involved in airway defense against bacteria. The abnormalities in CF lung secretions will be discussed in the next section.

The CF respiratory tract is a highly diverse and complex ecosystem posing several challenges to inhabiting microorganisms in the process. These challenges include oxygen and nutrient limitation, antibiotics, competing microorganisms, changing lung pathophysiology and hyperactive immune response. In healthy individuals, while the upper airways present the anatomical component that may favor bacterial colonization, the lower airways present a more complex interplay of anatomical variations with other factors such as oxygen availability and exaggerated immune response to invading microbes. The CFTR defect in CF changes the airway environment and anatomical parameters in the lower airways. The CF lower airways host diverse microorganisms and pathogens that are usually absent in healthy individuals. In addition, the microorganisms including pathogens evolve in these conditions to give rise to persisters [65–67] and mutators [68–72] that are capable of long-term surviving and colonizing the otherwise harsh lung environment [73]. It is plausible that the complex anatomical and physiological environments make CF lung a distinctive ecosystem with various niches that are eventually occupied by fitting microorganisms. This is supported by the different microbiota observed in different parts of the airways [74], and intrapulmonary spread of pathogens to previously unaffected niches [75]. The presence of active subpopulations of bacteria in particular areas of the airways is suggested to be potentially involved in pulmonary exacerbations in CF [76].

#### Cystic fibrosis sputum

The mucociliary system consists of the cilia, the mucus layer covering the airway and the airway surface liquid (ASL) layer. This mucus layer is separated from the underlying ciliated epithelium by the liquid phase ASL. The mucus forms a trap for bacteria, viruses, as well as other particles and molecules inhaled during respiration, and the ciliary beating carries them back to the pharynx by forming a “mucus escalator” where they are normally swallowed. Mucus production is thus an innate defense mechanism, which protects airway surfaces against irritants and infecting microorganisms. Mucus composition and viscoelastic properties are good indicators of pulmonary health [77]. Lamblin correctly reviews mucus as “an interface between the environment and the *milieu interieur*”.

In normal airways, if the ASL thins beyond a critical point, the surface epithelium converts from absorptive to secretory, though the exact mechanism is unclear. In CF, the defect in CFTR function leads to further absorption of isotonic liquid from ASL, leading to increase in thinning and viscosity of ASL. Thus, the gel-forming mucins that would otherwise float above the cilia are brought into close contact with the airway surface and attach to it. The antimicrobials within these mucus plaques soon become ineffective and invading microbes proliferate. The viscous mucous impairs ciliary beating, resulting in pulmonary ciliary dyskinesis, which results in the formation of mucous plaques. These mucus plaques, along with infecting microorganisms and resulting airway inflammation, lead to a decline in lung function. Conditions within the plaques, such as low O_2_ tension, have been shown to contribute further to the airway colonization by pathogens such as *P. aeruginosa* and *Streptococcus* spp*.* [78, 79].

Normal mucus is about 95–98% water and 2–5% of mucins with other materials. Mucins form a part of the airway innate immunity and play a very critical role in CF disease progression and treatment outcomes but also remain one of the poorly understood aspects of CF. In CF, the mucin to water ratio is about 5–10 fold higher than normal, with mucus viscoelasticity 10^4^–10^5^ fold greater than water at shear rates comparable to rubber [80]. Human airway mucins comprise a very broad family of high molecular weight glycoproteins. Structurally, mucins contain anywhere from one to several hundred carbohydrate chains attached to the peptide by O-glycosidic linkages between N-acetylgalactosamine and a hydroxylated amino acid. Very often, the carbohydrate chains are clustered in glycosylated domains. Apomucins, which correspond to their peptide part, are encoded by at least six different genes (MUC1, MUC2, MUC4, MUC5B, MUC5AC and MUC7). However, the carbohydrate chains that cover these peptides are highly variable. Given the structural diversity of these carbohydrates as well as their location at airway surfaces, mucins may be involved in interactions with inhabiting microorganisms. The expression of at least two of these genes (MUC2 and MUC5AC) have been shown to be inducible by bacterial products, tobacco smoke and different cytokines [77].

The ASL in CF lungs of children has been shown to be abnormally acidic [81]. Alaiwa et al. [81], in 2014 found that in neonates with CF, nasal ASL (pH 5.2 ± 0.3) was more acidic than in non-CF neonates (pH 6.4 ± 0.2). Opinions vary about whether ASL pH remains abnormally acidic with age [82]. In CF mouse model the decreased HCO_3_^−^ secretion due to CFTR defect and the unchecked H^+^ secretion by the non-gastric H^+^/K^+^ adenosine triphosphatase (ATP12A) acidifies ASL [50]. Experiments using CF primary airway epithelial cells stimulated with forskolin and 3-isobutyl-1-methylxanthine demonstrated that between HCO_3_^−^ and pH, it is the pH that affects the ASL viscosity more significantly [83]. It was suggested that the decreased pH probably affected di-sulfide bonds in mucins, thus stabilizing them and resulting in increased viscosity. More importantly, the acidification of ASL impairs airway host defenses, allowing microorganisms to thrive in the CF lung [50, 84].

Micro-rheological properties of CF sputum [85] have been investigated using techniques such as diffusion rates or behavior of a tracer (molecules, peptides, nanospheres and microspheres) using fluorescence recovery after photobleaching, dynamic light scattering, fluorescence correlation spectroscopy and single-particle tracking in multi-particle tracking experiments [86]. These experiments and biochemical analyses have demonstrated that sputum microstructure is significantly altered by elevated mucin and extracellular DNA content. Apart from bacterial infection, the presence of airway proteases and airway remodeling, which occur as the disease progresses, may also affect mucus properties and further alter mucociliary transport and bring about pulmonary inflammation. As CF lung disease progresses, the sticky mucus formed generates microaerobic or even anaerobic settings within the normally aerobic environment [87]. Such a lung environment of reduced oxygen level contributes to persistence of infection and decline of lung function [88, 89].

In addition to being an important factor in CF pathophysiology, CF sputum has been a key source of information on lung microbiome and disease state. Sputum analysis is a non-invasive alternative to bronchoalveolar lavage for obtaining airway secretions and has proven to be a reproducible and practical method for assessing airway inflammation and infection in adults.

### The airway microbiota and *Pseudomonas aeruginosa*

#### *Pseudomonas aeruginosa* in cystic fibrosis lung disease

*P. aeruginosa* colonization of the airways and infection remain the most important contributor to CF morbidity and mortality. While the CFTR defect results in myriad respiratory problems for the patient, the most important clinical feature is the chronic pulmonary infection with *P. aeruginosa*. Ultimately, more than 80% of patients with CF succumb to respiratory failure brought onto by chronic bacterial infection and concomitant airway inflammation.

*P. aeruginosa* is a Gram-negative aerobic to facultative anaerobic rod with a ubiquitous presence in the environment. It is well equipped with virulence systems such as toxin secretion systems for overcoming host defenses and inter-bacterial competition. It is capable of forming a well-organized bacterial consortium known as biofilm in the host. In CF, where the host immune response is compromised, *P. aeruginosa* presents itself as a dreadful threat and leads to a progressive decline in lung function.

There is still little clarity concerning acquisition of *Pseudomonas* in CF. Earlier studies pointed to clinical exposures and social interaction as zones for the acquisition of *P. aeruginosa*. These studies suggested that *P. aeruginosa* spreads by cross-infection [90–92]. The other identified risk factors in the acquisition of *P. aeruginosa* include gender, with females having more predilection than males, while F508del homozygous genotype and co-infection with other pathogens such as *S. aureus* and *B. cenocepacia* are also independent risk factors [93].

Despite having a low acquisition rate of just 1–2% per year, about 80% of patients are chronically colonized by *P. aeruginosa* by the age of 20 [94, 95]. In one study, the 8-year risk of death was found to be 2.6 times higher in patients having *P. aeruginosa* than in those without it [96]. Koch [97] suggested a “continuum for *P. aeruginosa* colonization” where the numbers of *P. aeruginosa* goes on increasing until there is “a point of no return”. The phase is marked by expression of biofilm forming genes in *P. aeruginosa* and appearance of host biomarkers such as antibodies against *P. aeruginosa*, an increase in polymorphonuclear leucocytes [PMN] and increased serine proteinases [97].

**Fig. 4:**
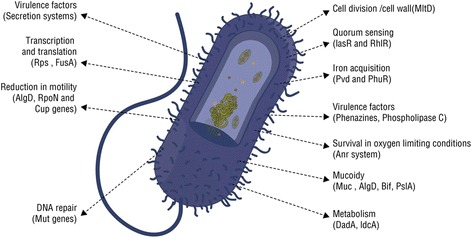
*P. aeruginosa* features relevant to pathogenicity and adaptation. *P. aeruginosa* produces an impressive array of virulence factors to counteract host defenses and facilitate inter-bacterial competition. The expression of virulence genes in *P. aeruginosa* is controlled by extremely complex regulatory circuits and signaling systems. The diagram outlines key features relevant to its pathogenicity and survival in vivo

*P. aeruginosa* must overcome challenges such as osmotic stress, competition from other colonizers, nutritional inadequacy, antibiotics, oxidative stresses, etc. in order to sustain and survive in the CF lungs. It characteristically overcomes these challenges by switching its gene expression [98]. During the course of CF lung colonization, it has been demonstrated that *P. aeruginosa* undergoes a life-style change to adapt to the CF environment. Chronic colonization is associated with genotypic and phenotypic changes in the bacterium such as increased antibiotic resistance, decreased metabolism, and slower growth rate, lack of motility, deficient quorum sensing and overproduction of alginate [Fig. 4] [90, 91, 93, 99–101]. Studies by others and by our laboratory have showed that *P. aeruginosa* switches from early acute infection to chronic, biofilm-associated infection by the activities of central regulatory systems such as the Gac-RsmA pathway [98, 102, 103]. A set of genes, especially those related to virulence factors and pathogenicity are turned on or off in response to the host environment to establish chronic infection. Preventing the chronic colonization of *P. aeruginosa* is very significant in avoiding associated lung function decline and development of resistance. Once established, *P. aeruginosa* chronic infection becomes almost impossible to eradicate although “seasonal” airway presence can take place with periods of re-infection and colonization [104]. The colonization may occur by the same strain, and in approximately 25% of the cases the colonization occurs by the similar genotype [105, 106]. In addition to gene expression switches, another explanation for changes in *P. aeruginosa* is the occurrence of parallel subpopulations or “hyper-mutator” strains, the mechanism for which is not well understood.

In response to the infection by *P. aeruginosa,* the polymorphonuclear leukocytes (PMNs) release reactive oxygen species (ROS) [107] and reactive nitrogen intermediates which, if able to overwhelm the infecting organism, damage the lipids and proteins inside it. However, if unable to do so, mutations in *P. aeruginosa* will allow for selection of the variants able to sustain these challenges [108]. One of the most common mutations occurs in *muc*A encoding anti-σ-factor that results in mucoid strains [109]. MucA limits the expression of the alginate (*algD)* operon through sequestering RNA polymerase σ factor σ^22^ encoded by *algU*. σ^22^ regulates stress response and is involved in regulation of virulence and motility in *P. aeruginosa* [107].

Among the other environmental stresses, CF patients often are exposed to a wide variety of antibiotics; thus adaptive resistance to antibiotics is common in *P. aeruginosa* [110]. Interestingly, many genes and regulatory systems involved in antibiotic resistance are often linked to regulation of other genes such as virulence-associated genes as well. Resistance-Nodulation-Cell Division (RND) pumps play important roles in *P. aeruginosa* resistance to antibiotics. There are 12 RND pumps present in the *P. aeruginosa* PAO1 genome. Results from our lab demonstrated that RND pumps such as MuxABC-OpmB and MexXY-OprM are linked, not only to antimicrobial resistance but to pathogenesis as well in *P. aeruginosa* [107]. PA0011 in PAO1 was found to be a regulatory gene involved in both carbapenem resistance and virulence in a temperature-regulated manner [107].

Genotypic and phenotypic variants within the *P. aeruginosa* population in CF lung may exist that contribute to different aspects of disease progression and pulmonary exacerbation. Due to the different conditions present in different areas of the respiratory tract, many variants of the same infecting strain may exist and heterogeneity within one population is common in the CF lung [111–113]. The heterogeneity manifests both in terms of genetic and physiological diversity and spatial distributions in the airways. This heterogeneous *P. aeruginosa* population harbors persisters and mutators which are very different from wild type cells and directly complicate therapeutic treatments. The activity and spread of certain subpopulations distributed in the airway niches are implicated in pulmonary exacerbations in CF [75, 76].

Though many aspects of chronic colonization by *P. aeruginosa* have been studied, others remain unanswered as to how *P. aeruginosa* overcomes competition by other bacteria to gain predominance? Do the other inhabiting bacteria support or compete with *P. aeruginosa*? What is the role of biofilm dispersal in infection state and disease? An in-depth knowledge of *P. aeruginosa* colonization mechanisms is critical to inform new therapeutic interventions.

#### *Pseudomonas aeruginosa* interaction with other bacteria

Until recently, individual pathogens, such as *P. aeruginosa* and *S. aureus* have been held responsible for CF lung infection and the resulting lung function deterioration. Previous studies in which the senior author of this review participated have shown that the interactions between *P. aeruginosa*, and avirulent oropharyngeal flora bacteria play important roles in disease pathophysiology [114]. Using genome-wide transcriptional analysis, changed expression of around 4% of genes in *P. aeruginosa* has been observed in the presence of some other members of the microbiota [115]. Subsequent studies have revealed that previously overlooked bacteria such as *Streptococcus milleri* group bacteria can cause exacerbations and lung damage in synergy with *P. aeruginosa* [115, 116]. Now, CF lung infection has been viewed as a polymicrobial infection and *Pseudomonas* and *S. aureus* co-infection and competition have been investigated widely in many studies.

It has been shown that colonizing *P. aeruginosa* triggers host cells to produce type-IIA-secreted phospholipase A2, a host enzyme with bactericidal activity capable of inhibiting *S. aureus* [117]. Using co-cultures of *P. aeruginosa* and *S. aureus* on bronchial epithelial cells homozygous for F508del-CFTR, Filkins et al. found that *P. aeruginosa* drives *S. aureus* from aerobic respiration to fermentative metabolism and reduces *S. aureus* viability. This eventually results in the predominance of *P. aeruginosa* in the community [118]. Nguyen et al. [119] recently demonstrated that *P. aeruginosa* not only inhibits the growth of *S. aureus* by regulating free iron levels, but also generates specific 2-alkyl-4 (1H)-quinolones, a class of antimicrobial compounds capable of lysing *S. aureus.* On the other hand, *S. aureus* can alter the host immune response to *P. aeruginosa*. It has been observed that *S. aureus* significantly inhibited the IL-8 production stimulated by *P. aeruginosa* and dampened Toll-like receptors (TLR1/TLR2)-mediated activation of the NF-κB pathway, highlighting the altered inflammatory response in polymicrobial infection [120].

*B. cenocepacia* infection in CF has been associated with poor prognosis and higher fatalities*.* Studies have shown that cis-2-dodecenoic acid produced by *B. cenocepacia*, also referred to as a *B. cenocepacia* diffusible signal factor, mediates the cross-talk between *P. aeruginosa* and *B. cenocepacia* by interference with quorum sensing systems and type three secretion system [121]. *P. aeruginosa* harbours genome islands with genes that are highly homologous to those found in *Burkholderia* sp. suggesting that there is a possibility of exchange of genetic material between the two organisms.

Viral infections are associated with pulmonary function decline, antibiotic use, prolonged hospitalizations and increased respiratory symptoms [122]. Respiratory syncytial virus is one of the most common viral co-pathogens in CF [123]. In association with *P. aeruginosa,* respiratory syncytial virus co-infection has been shown to aid *P. aeruginosa* colonization in CF patients [124].

Besides interactions directly affecting pathogenicity and host-pathogen relation*, P. aeruginosa* also interacts with other bacteria in the traditional sense of competition, antagonism and symbiosis affecting the structure and function of the airway microbiota. Iron is essential for both host and inhabiting pathogens, and complex systems of acquisition and utilization have evolved in microorganisms. Nutrient iron is tightly controlled by the host through complicated uptake, storage, and utilization systems, which also serve as a defense mechanism. To sequester iron, the microbes use specific iron-binding molecules known as siderophores [125]. *P. aeruginosa* not only can produce siderophores e.g. pyoverdine and pyochelin and use them to capture exogenous iron, but also can seize iron-bound mycobactin J, a siderophore produced by *Mycobacterium smegmatis* [126].

Type VI secretion systems (T6SSs) in *P. aeruginosa* are newly identified contractile nanomachines that transfer effector proteins across eukaryotic and prokaryotic cells and play a pivotal role in *P. aeruginosa* pathogenesis and inter-species competition [127, 128]. The first indication that T6SS could be involved in inter-bacterial interactions came from the identification of three effector proteins that are secreted by the hemolysin co-regulated protein secretion island-I-encoded T6SS of *P. aeruginosa* (H1-T6SS) [129]. Each of these three secreted effectors has toxicity towards other bacteria and is encoded adjacent to a gene encoding a product that provides immunity to the toxin, thereby preventing self-intoxication [130]. The effectors can be translocated between bacteria through the T6SS and provide a significant fitness advantage to the donor strain [131].

Clearly, dynamic interactions happen between the pathogens and other microbes in CF lungs. Such interactions not only affect the pathogenicity of the pathogen but also influence the host response, hence modulating the disease progression. Characterization of the complex microbial interactions within the CF airways is critical for understanding CF lung infection. Interesting questions hereafter arise: can the interaction/communication between different pathogens and other bacteria be used as a new antibacterial target and is it possible to manipulate such interaction to inhibit or disperse pathogens?

#### The cystic fibrosis airway microbiome

The earliest knowledge of CF airway microbiota depended on cultivability of the isolated microbes. Newer culture enrichment techniques enable gathering some of the missing information in the lung microbiota [132]. However, increasing studies use the non-culture-based, 16S rRNA metagenomic and meta-transcriptomic methods to decipher the complex microbiome in CF lungs. The earlier focus of respiratory microbiome studies was directed towards identifying bacterial members but later, investigations also characterized fungal and viral communities in CF and how interactions among these communities contributed to CF disease [133–136].

The airways present as a highly structured environment with varying niches which facilitates microbial diversity and fitness selection. Microorganisms adapting to such a dynamic environment can become either specialists or generalists for survival [108]. The microbiome of the lungs more closely resembles that of the oropharynx than the nasopharynx, and the gastrointestinal tract probably through hematogenous spread [137, 138]. Over 1000 microbial species (viruses, bacteria, moulds and fungi) have been identified in the airways of CF patients. Among them, bacteria typically made up more than 99% of the microbial community, while viruses and fungi constituted around 1% [139]. Although gut dysbiosis is an important feature of CF disease [140], the presence and composition of a symbiotic microbiome in human airways are still to be determined despite that recent researches suggested a disrupted respiratory microbiome in CF [141]. Significant associations have been discovered between age and diet and patterns of respiratory colonization, pointing to relations between intestinal microbiota, immune development, and respiratory microbiota in CF.

The pathogenic bacteria associated with CF include *P. aeruginosa, H. influenzae, S. aureus* and *B. cepacia* complex. Many studies have identified other taxa belonging to the genus *Prevotella*, *Streptococcus*, *Rothia*, *Actinomyces* and *Veillonella* as well. The emerging pathogens of clinical significance are listed in Table 1. Many of these often benignly colonize the upper respiratory tract (e.g., non-typeable *H. influenzae* or *S. aureus*) or are common environmental organisms that behave as pathogens only under certain conditions such as immunodeficiency.

**Table 1:** Emerging pathogenic species in CF respiratory microbiome

Species	References
*Stenotrophomonas maltophilia*	Waters et al., 2011 [209]
Methicillin resistant *Staphylococcus aureus* (MRSA)	Dasenbrook et al., 2008 [210] Chmiel et al., 2014 [211]
*Mycobacterium abscessus*	Malouf et al., 1999 [212] Binder et al., 2013 [213] Mahenthiralingam et al., 2014 [214]
*Achromobacter* spp.	Zemanick et al., 2011 [215] Tunney et al., 2008 [216]
*Streptococcus milleri*	Sibley et al., 2006 [116] Sibley et al., 2010 [217] Rabin et al., 2012 [218]
*Aspergillus fumigatus*	Sibley et al., 2006 [116] Stevens et al., 2016 [219] Speirs et al., 2012 [220]
*Nocardia* spp.	Heirali et al., 2016 [221]
Nontuberculous mycobacteria (NTM)	Caverly et al., 2016 [222]
*Scedosporium apiospermum* spp*.*	Parize et al.,2014 [146]
*Rasamsonia argillacea spp.*	Mouhajir et al., 2016 [148]
*Lomentospora prolificans* (*Scedosporium prolificans*)	Pihet M et al., 2009 [147]
*Exophiala dermatitidis*	Horré et al.,2010 [223]

**Fig. 5:**
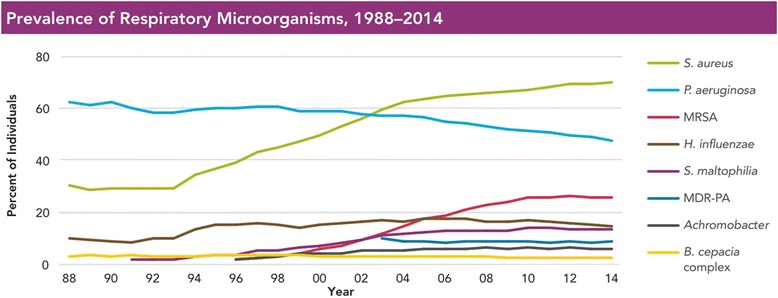
Prevalence of respiratory pathogens and antimicrobial resistant strains in patients with CF. As of 2003, *P. aeruginosa* is no longer the most common pathogen cultured in individuals with CF in USA. There has been an observable increase in the prevalence of *S. aureus* and *Strenotrophomonas maltophilia*. Figure reproduced with permision from Cystic Fibrosis Foundation Patient Registry, Cystic Fibrosis Foundation. Annual Data Report 2014. Bethesda, MD, USA [143]

**Fig. 6:**
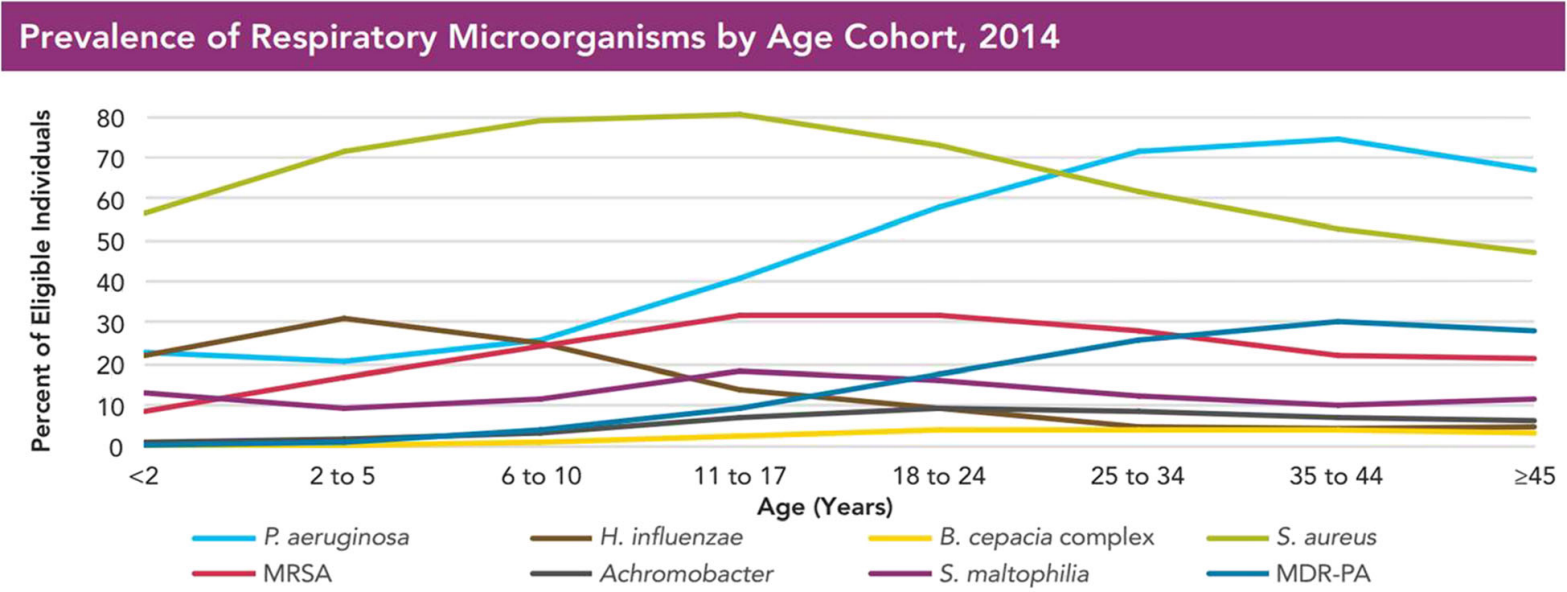
Prevalence of antimicrobial resistant strains in CF patients. An increase in the rates of multidrug-resistant *P. aeruginosa* infection has been observed in older CF patients in USA. Figure reproduced with permissions from the Cystic Fibrosis Foundation Patient Registry, Cystic Fibrosis Foundation. Annual Data Report 2014. Bethesda, MD, USA [143]

Interestingly, studies have found a trend of succession in the infecting organisms. *S. aureus* and *H. influenzae* are the most common bacteria isolated from the sputum in the first decade of life and *P. aeruginosa* is found to dominate numerically in the second and third decades of life [142]. This, however, is changing slowly [143] (Fig. 5), probably due to development of new therapies to control *P. aeruginosa* infection*.* The rate of multi-drug resistant *P. aeruginosa* in CF lungs, however, has been observed to be on a rise (Fig. 6). This suggests that *P. aeruginosa* can adapt well to incoming stressors, specifically antibiotics [142, 144].

Fungi and yeasts have also been identified as critical components of lung microbiome. Middleton et al. have reported associations between *Aspergillus* and *Candida* in the sputum of CF patients and worsened lung function. [145]. Given the small size of fungal spores, an inhalation allows easy access to bronchioles and alveoli where they can germinate and form hyphae. Poor clinical status seems to be associated with reduced fungal biodiversity and species richness [133]. *Aspergillus fumigatus*, species of the *Scedosporium apiospermum* complex [146], *Aspergillus terreus* and *Candida albicans* are commonly isolated from CF respiratory samples. Other fungal species including *A. flavus* and *A. nidulans* have been isolated transiently while *Exophiala dermatitidis* and *Lomentospora prolificans* (formerly *Scedosporium prolificans*) may chronically colonize the CF airways [147]. Species of the *Rasamsonia argillacea* complex (initially described as *Penicillium emersonii,* then as *Geosmithia argillacea* and finally reassigned to the genus *Rasamsonia*) [148] and *Acrophialophora fusispora* have been isolated almost exclusively in the context of CF. Interestingly, most fungal complications in CF patients have been known to be caused by filamentous fungi, which contribute to the local inflammatory response and cause progressive deterioration of the lung function [147].

Viruses, primarily phages that infect CF pathogens such as *Streptococcus*, *Burkholderia*, *Mycobacterium*, *Enterobacteria* or *Pseudomonas* species, have also been described in CF airways [149, 150]. Phages could serve as a means of horizontal transfer of resistance factors between different microbial species. Although no significant difference has been observed in the incidence of viral infections between CF and healthy controls, there is a marked difference in the severity and length of viral infections in patients with CF [151].

**Fig. 7:**
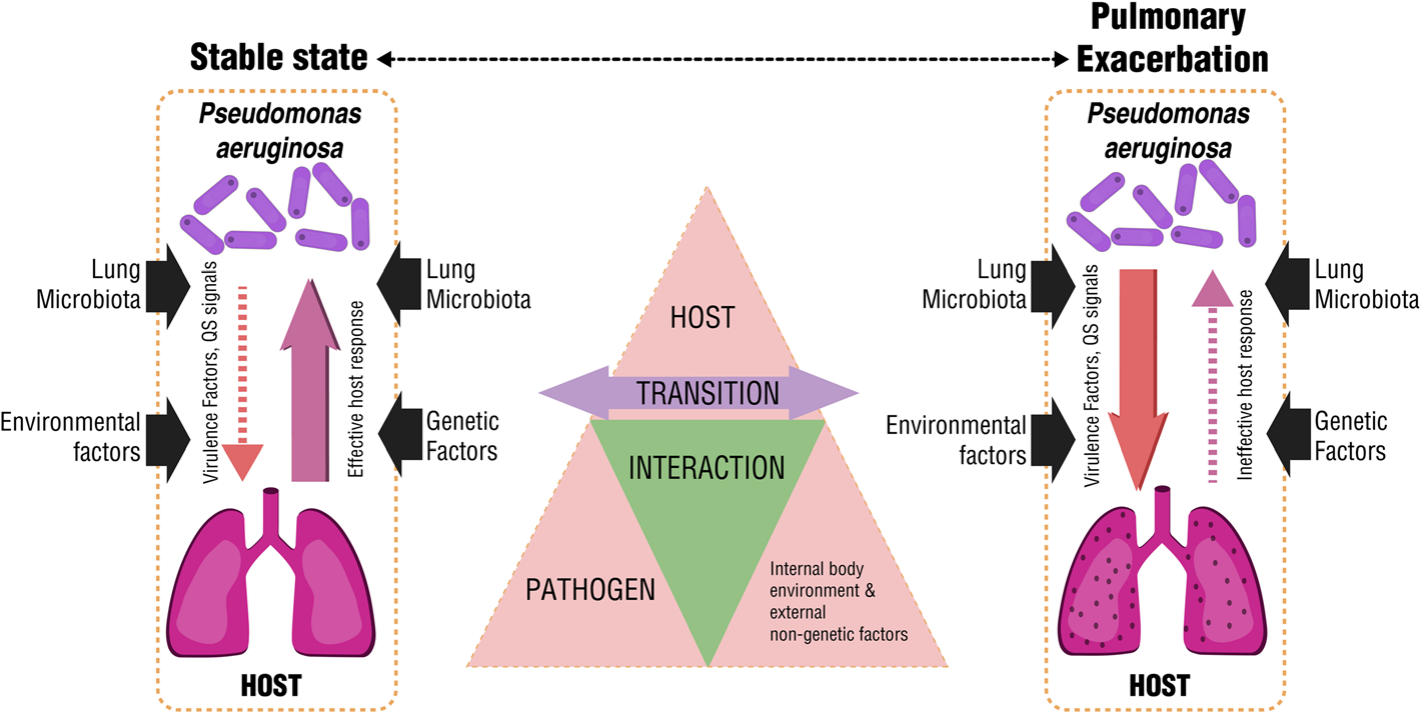
A potential mechanism of the transition from stable state to pulmonary exacerbation. Without much of a change in bacterial loads, the changes of the pathogenicity triggered by the host environment and/or host-microbiota interaction could lead to a transition from stable state to pulmonary exacerbation

There is no doubt that the progression of CF lung disease relates to CF respiratory microbiota. The complex microbiome challenges our understanding of pulmonary exacerbation and succession of infecting organisms. Fodor et al. [75] employed pyrosequencing to analyze the microbiota in CF patients and found that the microbial community composition was highly similar in patients during an exacerbation and when clinically stable. They suggested that intrapulmonary spread of infection rather than a change in microbial community composition may cause exacerbations [76]. However, a detailed study of day-to-day stability of the microbiome indicated that although the CF airway microbiota is relatively stable during periods of clinical stability, structural changes do occur which are associated with some, but not all, pulmonary exacerbations [152]. In other cases, an active subgroup of the lung microbiota may cause subtle changes in the microbiota which drive the onset of exacerbations [153]. It is possible that lung environmental alterations such as sub-inhibitory concentrations of antibiotics and host immune factors could cause changes in the virulence factors in the pathogens or changes in the functionality or metabolic activities of the microbiome, triggering exacerbations (Fig. 7).

Human health is a collective reflection of the human body and its associated microbiome. It is significant to review the factors that shape the CF lung microbiome. Although the lungs were classically believed to be sterile, recently published investigations have identified microbial communities in the lungs of healthy humans, and a strong association between markers of inflammatory lung diseases and bacterial community composition has been observed [154, 155]. Studies have observed a difference in lung microbiome from different climates, environmental microbiota, and even household pets. Apart from these factors, the CFTR genotype, the stage of disease and age [156] are among the factors that play a significant role in determining lung microbiome in CF. Alterations in lung environment including presence of antibiotics and changes in host immune responses alter not only bacterial diversity but also the metabolic profiles of inhabiting microflora [150, 157]. As many of the factors that shape the lung microbiome in CF patients differ in individuals the microbiome may be unique. Unlike microbiota in other part of our body, which is believed to have co-evolved with humans, CF lung microbiome could show even more diversities among individuals. Nevertheless, it can be expected that future investigation of the CF lung microbiome, especially its structural and functional changes over time, should improve our general understanding of CF, particularly with respect to disease progression, pulmonary exacerbation, host response and antibiotic therapies.

### Host immune response in cystic fibrosis lung

Efficient immune responses are required to protect the host from the harms of invading pathogens. Inflammation is tightly regulated in the host to avoid overshooting and collateral damage. CF lung disease is characterized by chronic and unresolved innate and adaptive responses in the infected airway compartments. Among innate immune cells, neutrophils are the first and predominant cell type transmigrating into CF airways. In adaptive immunity, T-helper cell type 2 and type 17 cell responses are predominant [158]; however, regulatory T cell responses are impaired in CF. Beyond these, the immune response in CF lungs is dysregulated at several levels, including impaired ceramide homeostasis, increased apoptosis, autophagy, and macrophage polarization to name a few amongst other deviations, as well discussed by Hart et al. [159].

**Fig. 8:**
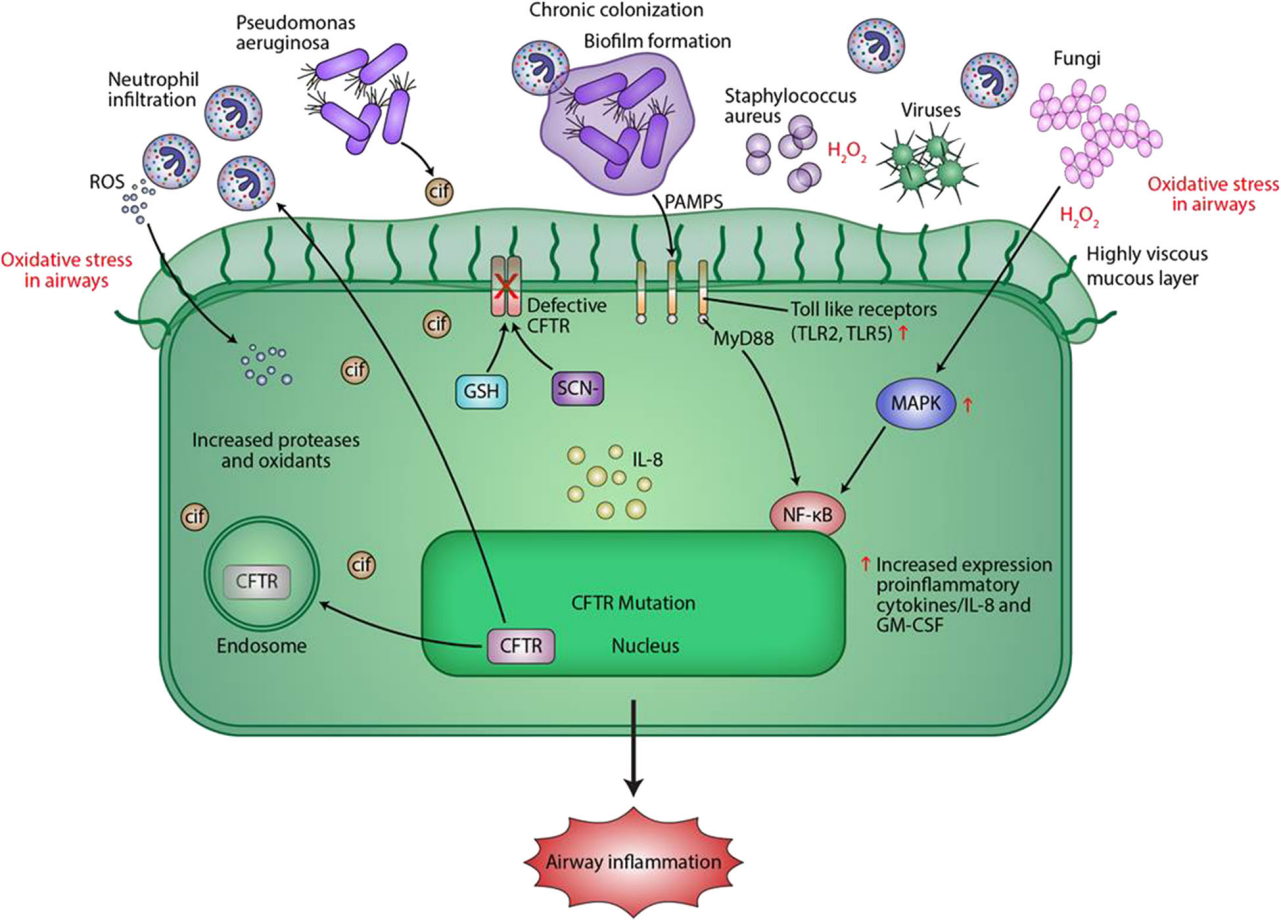
CF airway epithelium and pathogen adaptation. Defective CFTR leads to decreased airway surface liquid (ASL) layer. This facilitates microbial colonization and airway inflammation. Pathogen-associated molecular patterns (PAMPs) activate Toll-like receptor (TLR) signaling to activate Interleukin-8 (IL-8) and therefore to recruit polymorphonuclear neutrophils (PMNs). The increasing PMNs result in oxidative stress within the airways by forming reactive oxygen species (ROS). The increased oxidative stress further activates the mitogen activated protein kinase pathway, activating IL-8 and thus recruiting more PMNs. Mutated CFTR in the epithelial cells is unable to channel the antioxidants Glutathione (GSH) and thiocyanate (SCN^−^) into the airway, limiting their ability to counteract the oxidative stress. TLR expression and signaling is also altered in CF epithelium. Expression of TLR2 and TLR5 at the apical surface is increased, whereas TLR4 expression is limited to endosome (not shown here). NF-κB in CF airway epithelial cells is constitutively activated, resulting in the production of inflammatory cytokines including IL-8 and granulocyte macrophage colony stimulating factor (GM-CSF). This also leads to recruitment of PMNs independent of TLR’s interaction with the adaptor protein MyD88. Bacterial PAMPs further increase NF-κB signaling through activation of TLR-MyD88 signaling. The inhaled bacteria start interacting and aggregating to form biofilms. *P. aeruginosa* also releases outer membrane vesicles containing CF inhibitory factor (Cif), a protein that further inhibits the recycling of CFTR in the host further contributing to the cycle of hyper-inflammation and bacterial colonization

Phagocytic innate immune cells such as neutrophils and macrophages accumulate within the airway compartments. Unfortunately, they are unable to clear the infecting organisms effectively and the bacteria continue colonizing and forming biofilms (Fig. 8). Neutrophils in CF demonstrate imbalance in ion homeostasis with increased levels of chloride and sodium and decreased levels of magnesium. This results in impaired degranulation and defective phagocytosis [159]. In response to invading pathogens, particularly *P. aeruginosa*, neutrophils accumulate within airways and release powerful anti-microbial compounds, such as neutrophil elastases and myeloperoxidases. Along with nicotinamide adenine dinucleotide phosphate oxidase (also from neutrophils), these compounds result in production of reactive oxygen and nitrogen species. Mutated CFTR in the epithelial cells is unable to channel the antioxidants, glutathione [160] and thiocyanate [161] into the airways to counter the oxidative stress. The increased oxidative stress further activates pro-inflammatory cytokines such as IL-8, resulting in hyper-inflammation within the already inflamed airways.

Neutrophils also use their extracellular host defense armamentarium, also known as neutrophil extracellular traps (NETs) [162] and the intracellular stored anti-microbial effector proteins such as defensins and proteases for killing pathogens. NETs are composed of a DNA backbone with entangled histones and neutrophilic granular components. It is suggested that NETs may protect the host cells from unregulated protease activity [163]. Interestingly, NETs have been shown to be inducible by pyocyanin, a toxin and virulence factor secreted by *P. aeruginosa* in a time- and dose-dependent manner [164]. Thus, bacteria may use NET-mediated killing to exacerbate airway inflammation for their own benefit.

The accumulation of mucus in the airway is consequent to neutrophil elastase accumulation, which is a serine protease secreted by neutrophils in their azurophilic granules and macrophages under inflammation. Neutrophil elastase plays a role in degrading bacterial proteins in azurophilic granules. Under normal conditions, neutrophil elastase is blocked by anti-proteinases such as α1-antitrypsin, the secretory leukocyte peptidase inhibitor, and elafin. However, the amount of neutrophil elastase overcomes the blocking anti-proteinases in CF [165] which can harm pulmonary matrix. Because NE levels correspond positively to neutrophil counts, and IL-8, IL-1ß and matrix metalloproteinase-9 levels, they are considered a significant marker for predicting CF infection and diseases [153, 166]. In addition, studies have shown that neutrophil elastase is capable of degrading CFTR [165] as well as cell surface receptors such as CD2, CD4, CD8, complement receptor 1 and antigen presentation receptors.

Macrophages function as phagocytes, clearing both pathogens and apoptotic cells through efferocytosis. Macrophages can be classically subdivided into M1 and M2 macrophages. M2 cells are inducible in a T helper type 2 cell-responsive environment and are capable of causing fibrosis and extracellular matrix remodeling. Recognition of pathogens by macrophages is through TLRs. Among TLRs, *P. aeruginosa* has been reported to be sensed through TLR2, TLR4, TLR5 and/or TLR9. *P. aeruginosa* mutants defective in flagella were resistant to TLR activation and thus could survive airway macrophage phagocytosis and killing. TLR-4 is also expressed in neutrophils. It senses bacterial LPS and leads to lysosomal degradation of bacterial LPS. Descamps et al. [167] reported that TLR5 engagement is crucial for bacterial clearance by murine macrophages in vitro and in vivo*. P. aeruginosa* mutants defective in TLR5 activation via flagella were resistant to phagocytosis and killing by airway macrophages. Decreased expression and dysregulation of TLR-4 is found to be a factor for continuous inflammation in CF diseases [110, 168, 169].

In addition to TLRs, another class of strictly intra-cellular receptors has been described known as Nod-like receptors (NLRs) and NLR family apoptosis inhibitory protein (NAIP). These NLRs and NAIPs react with microbial pathogens-associated molecular patterns (PAMPs) on *P. aeruginosa* to form inflammasomes*.* NAIP subtypes NAIP-5 and NAIP-6 have been shown to interact with flagellin protein of *P. aeruginosa* [170], whereas the intracellular receptors NAIP2 and NLR family caspase recruitment domains containing protein 4 (NLRC4) also known as IPAF have been shown to interact with the inner rod protein of the type III secretion system in *P. aeruginosa* [171]. Pili from *P. aeruginosa* have been shown to activate the NLR and NAIP inflammasomes, independently of NLRC4, through an unknown mechanism [172–174]. Cif, a bacterial virulence factor secreted in outer membrane vesicles by *P. aeruginosa*, has been shown to increase the ubiquitination and lysosomal degradation of CFTRs. Bomberger et al. [175] have shown that Cif inhibits TAP (transporter associated antigen processing) and major histocompatibility complex (MHC) class I antigen presentation. In response to the PAMPs and damage-associated molecular patterns from the invading organisms, both epithelial cells and immune cells induce a pro-inflammatory cascade with high level secretion of cytokines and chemokines resulting in lung function decline and lung tissue damage.

Regulatory T cells (T_Reg_) are essential for preventing autoimmune diseases, limiting chronic inflammatory diseases, and maintaining immune homeostasis. A decreased in CD4+, CD25+, and FoxP3+ T cells in CF has been observed [169]. Invariant NKT (iNKT) are another special subset of T cells. These iNKT cells express an invariant T cell receptor chain (Vα24-Jα18 segments in humans) with a beta chain. They can identify glycolipid antigens such as CDId, a highly conserved non-polymorphic MHC class I-like molecule. It has been shown by Seigmann et al. [176] that CFTR deficiency in CF mouse models provokes a significant increase of iNKT cells in the lung. Thus, in CF lungs ceramide-mediated cell death results in activation of iNKT cells that drive the recruitment of inflammatory cells to the lung tissue, further promoting inflammation and lung tissue injury [176].

Myeloid-derived suppressor cells (MDSCs) balance innate and adaptive immunity by regulating T cell response in chronic illnesses. It is suggested that *P. aeruginosa* induces the production of MDSCs, and modulates T-cell defense in CF through flagellin [177]. MDSCs accumulate in CF patients with chronic *P. aeruginosa* infection, which may result in the inhibition of T cell generation and Th17 response, and hence in the protection of *P. aeruginosa* [177].

Human leucocyte antigens-DQ is an MHC class II molecule expressed on dendritic cells, monocytes and macrophages. In CF, there is a reduction in HLA-DQ expression on immune cells in blood and lung [177]. It is speculated that this may be a result of insufficient interferon response; however, the impact of this finding on the development of CF disease is still unknown.

Sputum club cell secretory proteins (CCSPs) are biomarkers of CF disease. These proteins produced by Clara cells possess anti-inflammatory qualities. Previously it was observed that CCSP expression and production are decreased in chronic asthmatic patients [178] and decreased levels of CCSP in broncho-alvelolar lavage fluid were also found in CF patients [179]. Patients with mucoid *P. aeruginosa* variants also had a significantly lower CCSP concentration in sputum. Readers are also referred to review by Cohen and Prince on CCSP [180].

In CF, the repetitive cycle of inflammatory cell recruitment and unregulated immune cell activation causes tissue damage and leads to irreversible loss of lung function. The defect CFTR undermines first line of host defense in the pulmonary system and activates an inflammatory cascade independent of infectious stimuli. Consequently, CF therapeutic intervention relies heavily on anti-inflammatory and anti-microbial drugs. However, only partial success for anti-inflammation in CF lung disease has been achieved so far. Recent understanding of the connection of human microbiome homeostasis and chronic inflammatory response may have provided another avenue to rebalance the host immune response in CF airways by targeting the CF lung microbiome or restoring a healthy overall human microbiome.

### External non-genetic influences in cystic fibrosis

Though non-genetic factors do not impact the disease pathogenesis directly, they do have an impact on overall outcome of the disease and in overall well-being of a CF patient. Nutrition, exercise, external environment, and psychology can all have significant impact on CF outcomes. Non-genetic influences have been estimated to account for approximately 50% of the clinical variation in CF [181]. Readers are referred to review by Schechter [182, 183] for detailed discussion on these factors. The impact of these factors on above discussed topics is briefly discussed here.

Nutritional status and lung function are highly correlated in CF, and inadequate nutrition is associated with decreased lung function and survival. The precise mechanism of this relationship is unknown but, it has been suggested that malnutrition may impair immunologic defenses against incoming infection and cause respiratory muscle weakness [184]. There may be a role for probiotics in promoting weight gain and controlling inflammatory response in CF children [185]. In retrospect, extensive antibiotic administration in CF may also contribute to weight loss and lethargy.

The hyper-inflammatory response to lung infection in CF is persistent and generally harmful for the host. Thus, external factors that contribute to the  inflammatory response may further damage the already compromised system. Environmental factors, such as exposure to particulate matter, have also been proven to play a role in pulmonary exacerbations and result in decline in the lung function, with ambient air quality for CF patients being especially relevant [186]. Roadside and freeway elements such as industrial pollutants, traffic pollutants, automobile tires, road dust and diesel exhaust have been proven to increase the lung inflammation by increasing the lung cytokines expression [187]. Environmental tobacco smoke is yet another factor which can cause respiratory exacerbation, lung growth impairment *in utero* and decline in lung function in adults with CF [187]. Cigarette smoke was first reported to inhibit chloride secretion in excised canine tracheas [188], and a dose-dependent association between tobacco smoke exposure and overall disease severity in CF was observed. Secondary smoke exposure is as harmful as firsthand smoking. In CF it is observed that secondary smoke exposure activates MEK/ERK pathway and disrupts CFTR expression and function [189]. Interestingly, elevated calcium concentration (observed in CF) has also been shown also to activate the MEK/ERK signaling pathway. Secondary smoke exposure has been reported to be associated with isolation of methicillin-resistant *S. aureus* and anaerobic bacteria in oropharyngeal cultures of infants below 12-months of age [190]; these pathogens could have an impact on the composition of lung microbiota in the case of CF patients.

Exercise has been shown to influence lung function positively in CF patients if carried out in a beneficial, safe and monitored environment [191, 192]. Spiritual factors interestingly have been shown to contribute positively on treatment adherence in CF patients [193]. Social behavior factors such as self-esteem and social stigma can also be associated with pulmonary function in CF [194]. Studies have demonstrated the prevalence of anxiety and depression in patients with CF and their parents to be as high as 30%, significantly higher than in the general population [195, 196]. It has been shown that there are reciprocal connections between a host’s social behavior and its microbiome. Considering that social behavior can alter microbiome composition by affecting transmission, it becomes not far-fetched to connect such factors to CF airway microbiome or lung infection. Non-genetic factors can influence the disease outcome; thus, it is important to understand these and intervene accordingly.

### Update on therapeutic options

The last 35 years have seen exciting new developments in antimicrobials, small molecule correctors and CFTR expression enhancers for CF lung disease. However, tackling lung function impairment in CF patients has remained a challenge, and continuing effectors towards either restoring host function (CFTR function and mucociliary clearance) or controlling disease progression (inflammation and infection) are required. Table 2 outlines some of the recent therapeutic approaches in managing CF disease. The classical approach in management of CF involves chest physiotherapy, oxygen therapy, nutritional support, prophylactic and interventional therapy. Bronchodilators such as beta-adrenergic agonists, anticholinergic drugs, and/or theophylline have been shown to help patients with mild overall lung disease. Mucolytics such as dornase alfa and hypertonic saline inhalation have been shown to benefit in CF by increasing hydration of ASL in patients. Corticosteroids have shown several important effects on neutrophil-mediated inflammation; however, the risks involved must be dealt with.

**Table 2:** List of new therapies in CF

Type of therapy	Name of the therapy	Benefits of the therapy	References
Airway clearance therapy	Dornase-alfa	Breakdown of excess DNA in cell debris and mucous of chronically inflamed airways	Mogayzel et al., 2013 [224]
	Nebulization of hypertonic saline	Increases airway hydration and mucociliary clearance	Donaldson et al., 2006 [225]
Antibiotics therapy	Tobramycin	Helpful in chronic stages of cystic fibrosis	Mogayzel et al., 2013 [224]
	Aztreonam lysinate	Improves lung function and reduces incidences of pulmonary exacerbations	Assael et al., 2013 [226]
	Colistin (colistimethate sodium)	Improves lung function	Schuster et al., 2013 [227]
Anti-Inflammatory therapy	Azithromycin	Macrolide antibiotic. Long term usage in CF reduces neutrophilic inflammation and pulmonary exacerbations. Improves lung function	Uzun et al., 2014 [228] Saiman et al., 2010 [229]
	Ibuprofen	Reduces the decline in lung function. Especially effective in pediatric patients	Lands et al., 2007 [230] Mogayzel et al., 2013 [224]
Gene therapy	1.Zinc finger nucleases 2.Transcription activator –like effector nucleases (TALENs) 3.RNA guide engineered nucleases (derived from CRISPR)	These nucleases cleave the DNA at a specific site of interest and allow genomic modifications	De Boeck et al., 2014 [231] Dekkers et al., 2012 [232]
CFTR modulation	1.Potentiators (VX-770, VX661 Ivacaftor, Lumacaftor, Riociguat, QBW251). 2.PTC therapeutics or ataluren	Aimed at correcting the dysfunction of CFTR like nonsense, frameshift, splice mutations and non-functional CFTR mutations.	Van Goor et al., 2009 [233] Du et al., 2008 62 62 [234]

Newer therapies include the use of human mesenchymal stem cells (hMSCs) which are known for their anti-inflammatory and antibiotic properties. An in-vivo study on CF lung treated with hMSCs showed significant reduction in the bacterial infection and an increase in the production of antimicrobial peptide LL-37. hMSCs have the potential to increase the efficacy of antibiotics and decrease the bacterial growth by releasing soluble products [197]. In addition to the bronchial epithelial cells, nasal airway epithelial cells, intestinal organoids from intestinal stem cell biopsy, monocytes and macrophages have also been studied for CFTR therapy [198, 199].

Genome editing has played an important role in CF studies and it also presents as a promising therapeutic approach. Triplex-forming peptide nucleic acid molecules delivered in nanoparticles have been used successfully to correct F508del CFTR in airway epithelium in vitro [200]. mRNA level editing using oligonucleotides to correct F508del has been evaluated clinically [201]. Gene editing using transcription activator-like effector nucleases (TALENs) or the clustered regularly interspaced short palindromic repeats (CRISPR/Cas9) system has also been explored as a strategy to correct CFTR mutations. However, the exact way in which this might be administered remains to be investigated as it may be argued that the target cells are buried beneath the surface epithelium and may be difficult to access with gene therapy vectors [201–203].

The CFTR potentiator Ivicaftor approved by the Food and Drug Administration in 2012 is an exciting new drug that restores the channel function of CFTR. The drug improves pulmonary functions along with mucociliary clearance and exacerbation in CF patients [204]. Ivacaftor is used for patients with specific CFTR mutations including G551D and several other mutations [204].

Recent years have also seen much advancement in development of new antimicrobials or novel delivery approaches. Leucocytes of Old World monkeys produce cyclic cationic peptides called θ-defensins. Rhesus θ-defensin-1 is known to exhibit anti-bacterial properties against *P. aeruginosa*. A study conducted on a CFTR F508del-homozygous murine model of chronic *P. aeruginosa* lung infection indicated that treatment with Rhesus θ-defensin-1 significantly decreased lung infection and airway neutrophils [205]. Azithromycin-loaded and rapamycin-loaded nanocomposite microparticles a dry-powder-based antibiotic therapy, has been shown to enhance pulmonary antibiotic delivery [205, 206]. Recently, the use of phages, bacteria-specific viruses that kill pathogens has emerged as a promising alternative. To deal with the problem of pathogenic bacteria developing resistance to phages, pre-adapting phages to bacterial pathogens is used to improve the efficacy of phage therapy both by reducing phage resistance as well as by increasing the phage infectivity. Phage-therapy with pre-adapted or evolved phages have been shown to efficiently decreased the bacterial density of chronic isolates in comparison with the ancestral phages [207]. Phages are easy to develop and can be very specific whereas phage-resistant bacteria remain susceptible to other phages with a similar target range.

Despite all the advances, no isolated therapy is completely effective; combination therapy on the other hand has demonstrated far better efficacy. For example, Lumafactor combined with Ivacaftor improves forced expiratory volume (FEV_1_) and reduces pulmonary exacerbations in F508del CFTR model [208]. In addition to new drugs, integration of comprehensive therapies, technologies, and disease management that are based on holistic understanding of CF pathophysiology, are required.

## Conclusions

*Pseudomonas aeruginosa* chronic infection is perhaps the most serious problem in CF lung disease. Exploring the pulmonary environment and physiological deviations in CF is valuable in understanding disease pathogenesis. Though we now recognize CF as a polymicrobial disease, better understanding of the CF lung disease requires investigating the CF airway microbiome, the interactions among the pathogens, the host, the environment and the resulting immune response. The growing knowledge on the complexity of CF airways and the emerging role of sinuses in bacterial persistence has opened up new targets for anti-microbial therapy and thus limiting the chronic infections. We now also know that overshooting of inflammation in CF can support facultative anaerobes, such as *E. coli* and *P. aeruginosa* in inflamed respiratory tracts. Further investigation of bacterial pathogenicity and its regulatory systems, as well as the relationship between host responses and human microbiome should provide novel approaches to control infections in CF. New techniques in enhancing mucociliary clearance and restoring CFTR function will also bring CF patients more effective treatment options.

In addition, pre-term screening, early diagnosis and intervention, counselling, better nutrition, exercise, maintaining favorable external environmental factors such as clean ambient air and limited exposure to potential infection at public places, the availability of multi-speciality healthcare systems should help slowing the progression of CF. Big leaps may be expected soon in terms of improving patient health and life.

## Abbreviations

ASL: Airway surface liquid
ATP: Adenosine tri-phosphate
cAMP: Cyclic adenosine monophosphate
CCSP: Club cell secretory protein
CF: Cystic fibrosis
CFTR: Cystic fibrosis transmembrane conductance regulator
hMSC: Human mesenchymal stem cell
ICL: Intracellular loop
IL: Interleukin
iNKT: Invariant natural killer cells
LPS: Lipopolysaccharide
MDSC: Myeloid-derived suppressor cell
MHC: Major histocompatibility complex
NAIP: Neuronal apoptosis inhibitory protein
NBD: Nuclear-binding domain
NET: Neutrophil extracellular trap
NLR: Nod-like receptor
NLRC4: NLR family CARD domain containing protein 4
PAMP: Pathogen-associated molecular pattern
PMN: Polymorphonuclear leukocytes
RND: Resistance-nodulation-cell division
ROS: Reactive oxygen species
T6SS: Type six secretion system
TLR: Toll-like receptor
